# Preoperative Diagnosis of Usual Leiomyoma, Atypical Leiomyoma, and Leiomyosarcoma

**DOI:** 10.1155/2014/498682

**Published:** 2014-10-21

**Authors:** M. Matsuda, T. Ichimura, M. Kasai, M. Murakami, N. Kawamura, T. Hayashi, T. Sumi

**Affiliations:** ^1^Department of Obstetrics and Gynecology, Osaka City University Graduate School of Medicine, 1-5-7 Asahimachi, Abeno-ku, Osaka 545-8585, Japan; ^2^Department of Obstetrics and Gynecology, Osaka City General Hospital, Osaka 534-0021, Japan; ^3^Department of Immunology and Infectious Disease, Shinshu University Graduate School of Medicine, Nagano 390-8621, Japan

## Abstract

Uterine smooth muscle tumors (SMTs) are common pelvic tumors in women, and most of them are diagnosed as usual leiomyoma (UL). Exclusion of malignant disease is important in the management of SMTs. However, differentiation of SMTs remains difficult. In this study, we aimed to improve the preoperative diagnosis of SMTs. We examined 21 ULs, 7 atypical leiomyomas (ALs), and 6 leiomyosarcomas (LMSs), all of which were diagnosed by uterine tumor biopsy. Immunohistochemical findings (low-molecular-mass polypeptide 2 (LMP2) and Ki-67) and clinical features (serum lactate dehydrogenase level and menopause) were evaluated. Statistically significant differences in the expression of LMP2 and Ki-67 were observed between UL and AL and between UL and LMS. The combined LMP2 and Ki-67 score was significantly different between UL and AL, between UL and LMS, and between AL and LMS. The combined immunohistochemistry and clinical findings score (total score) was also significantly different between pathological types. The findings of this study suggest that the accuracy of the preoperative diagnosis of SMTs may be improved by using a combination of immunohistochemical and clinical findings.

## 1. Introduction

Uterine smooth muscle tumors (SMTs) are common pelvic tumors in women, and most of them are usual leiomyomas (ULs). Leiomyosarcoma (LMS) is rare, constituting only about 1% of uterine cancers; however, it has a poor prognosis [1]. Diagnosis of LMS depends on the presence of cytologic atypia, a high mitotic index (MI), and coagulative tumor cell necrosis (CTCN). Some cases partially have these features, which helps to distinguish between UL and other SMTs. Atypical leiomyoma (AL) is one such SMT, and its pathological features lie between those of LMS and UL; however, its degree of malignancy is uncertain [2]. Although most LMSs arise in postmenopausal women, several cases have been reported in women of reproductive age [1, 3]. Patients with AL are younger than those with LMS and often desire to have children [4].

Various treatments are available for the management of symptomatic UL. Uterus-preserving treatments such as myomectomy, administration of gonadotropin-releasing hormone analog, uterine arterial embolization, and focused ultrasound surgery are widely performed. With the exception of myomectomy, surgical specimens are not obtained using these techniques, and a pathological diagnosis is impossible. Therefore, pretreatment differentiation of SMTs is important. Imaging modalities, especially magnetic resonance imaging (MRI) and fluorodeoxyglucose- (FDG-) positron emission tomography, are useful for the diagnosis of SMTs. However, distinguishing among the different types of SMT remains difficult in many cases [5, 6].

Since 1994, we have performed transcervical needle biopsy to improve the preoperative differential diagnosis of SMTs [7]. Although this procedure enables us to directly evaluate the tumor tissues, the biopsy specimens are very small and the diagnosis may be underestimated. We have attempted to improve the preoperative diagnosis using immunohistochemistry (IHC). We previously reported that the expression of Ki-67 in LMS was higher than that in UL using biopsy specimens [8].

A recent study reported that defective expression of low-molecular-mass polypeptide 2 (LMP2) may initiate the development of spontaneous human uterine LMS [9]. LMP2 is encoded in the major histocompatibility complex class region of the 20S proteasome, which is part of the 26s complex that degrades ubiquitin-conjugated proteins [10]. LMP knockout mice were shown to develop uterine LMS [9].

To improve the quality of preoperative diagnosis of SMTs, we performed a retrospective evaluation of the clinical and IHC features of SMTs by anti-LMP2 and Ki-67 examination of specimens obtained by transcervical needle biopsy.

## 2. Materials and Methods

### 2.1. Case Selection

We performed a search of all inpatient files from the Department of Gynecology at Osaka City University Hospital for all patients with uterine SMTs with available needle biopsy specimens in the years 2000 and 2013. All patients underwent transcervical needle biopsy because of unusual MRI findings (high intensity on T1- and/or T2-weighted images) or a rapidly enlarging tumor. Thirty-four cases of SMT were found: 6 cases of LMS, 7 of AL, and 21 of UL. AL was defined as an SMT with moderate to severe atypia, an MI of <10 per 10 high-power fields, and no CTCN.

All tissues were used with the approval of the Ethics Committee of Osaka City University after written informed consent had been obtained from the patients.

### 2.2. Transcervical Needle Biopsy of Uterine SMTs

Transcervical needle biopsy of uterine SMTs was performed under transabdominal guidance using a Pro-Mag 2.2 biopsy system (Manan Medical Products, Northbrook, IL) with an automatic cutting needle (25 cm long, 16-gauge, and 17 mm notch) and a 20 cm long straight stainless steel guide pipe (4 mm maximum external dimension, 3 mm maximum internal dimension, Honest Medical, Tokyo, Japan). To prevent sampling error, three or more biopsy specimens per patient were obtained [7].

### 2.3. IHC Staining Procedures

Expression of LMP2 and Ki-67 was investigated in paraffin-embedded sections using the avidin-biotin peroxidase complex method. The 4 *μ*m thick paraffin sections were deparaffinized and immersed in 3% hydrogen peroxidase to block endogenous peroxidase activity. Next, an antigen retrieval procedure was performed only for the sections undergoing examination of Ki-67 expression by immersing the slides in 10 mM citrate buffer (pH 6.0) and heating in an autoclave at 120°C for 20 min. The protocol for the DAKO LSAB 2 peroxidase kit (DAKO, Kyoto, Japan) was followed. The sections undergoing examination of Ki-67 expression were incubated overnight with the primary antibody in a humidified chamber at 4°C, and the sections undergoing examination of LMP2 expression were incubated for 1 hour with primary antibody in a humidified chamber at room temperature. The primary antibodies used for this study were monoclonal mouse anti-human Ki-67 and polyclonal rabbit antiproteasome 20s LMP2. The working dilutions for each primary antibody were 1 : 20 for anti-Ki-67 and 1 : 100 for anti-LMP2. Sections were rinsed with PBS three times and incubated for 10 min with the secondary antibody (biotinylated goat anti-mouse and rabbit immunoglobulin G secondary antibody). The sections were then incubated with streptavidin-peroxidase complex, and 3,3′-diaminobenzidine was used as a chromogen. The sections were counterstained with Mayer's hematoxylin. The specificity of the IHC reactions was checked by omitting the primary antibody [8].

### 2.4. IHC Scoring

LMP2 expression positivity was indicated by brown-staining cytoplasm. The staining intensity was scored as positive = 2 points, weakly positive = 1 point, and negative = 0 points. The stained area was classified as negative = 0 points, focal = 1 point, and diffuse = 2 points (Table 1(a)).

Expression of Ki-67 was evaluated using the labeling index (LI). We counted the positive cells with a brown-stained nucleus at high magnification (×40 objective and ×10 ocular); an LI of ≤5% was assigned 2 points, an LI of >5 to <15% was assigned 1 point, and an LI of ≥15% was assigned 0 points (Table 1(b)). We observed all the areas of all cores and then chose the most active area and calculated LI for each case.

Finally, we calculated the product of each score as the IHC score. The product of the IHC and clinical scores was defined as the total score.

### 2.5. Clinical Features

We also evaluated each patient's serum lactate dehydrogenase (LD) level and menstrual situation. For the serum LD level, we calculated the LD ratio as the maximum serum LD value/the upper limit of the normal range. When the LD ratio was >1.2, we determined that the patient had a high serum LD level and assigned a score of 0 points (Table 2(a)). We assigned a score of 1 point for premenopausal patients (Table 2(b)).

### 2.6. Statistical Analysis

The Mann-Whitney* U* test was used to compare the scores among the analyzed groups (UL, AL, and LMS). *P* values of <0.05 were considered statistically significant. SPSS version 21 (IBM) was used for all calculations.

## 3. Results

### 3.1. Clinical Features

The patients' clinical features are summarized in Table 3. The patients' ages ranged from 31 to 69 years with a median age of 43 years in patients with UL, 46 years in patients with AL, and 56 years in patients with LMS. No patients took medication, hormones, and supplements. One, one, and three patients with UL, AL, and LMS were postmenopausal, respectively. A high LD level was recorded in only three patients with LMS. Three patients with UL and one with AL underwent myomectomy; the remaining patients underwent total hysterectomy. No patients with AL developed recurrence.

### 3.2. IHC Findings

**Figure 1:**
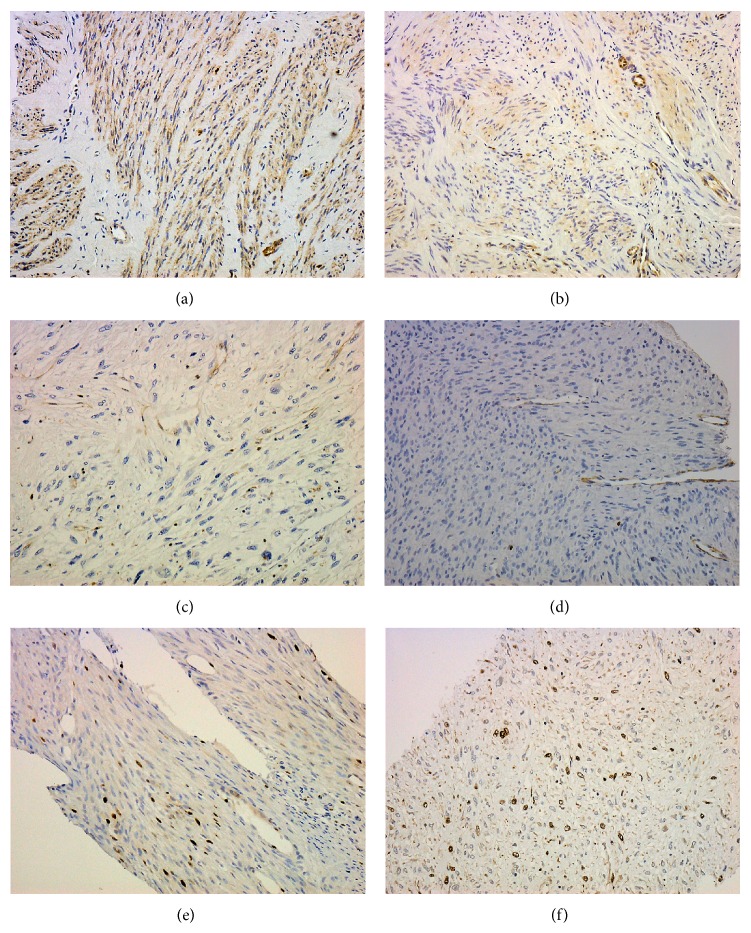
LMP2 and Ki-67 expression. (a) Positive expression of LMP2 in UL. (b) Weakly positive expression of LMP2 in UL. (c) Negative expression of LMP2 in LMS. (d) Ki-67 expression in UL (LI ≤ 5). (e) Ki-67 expression in UL (5 < LI < 15). (f) Ki-67 expression in LMS (LI ≥ 15).

**Figure 2:**
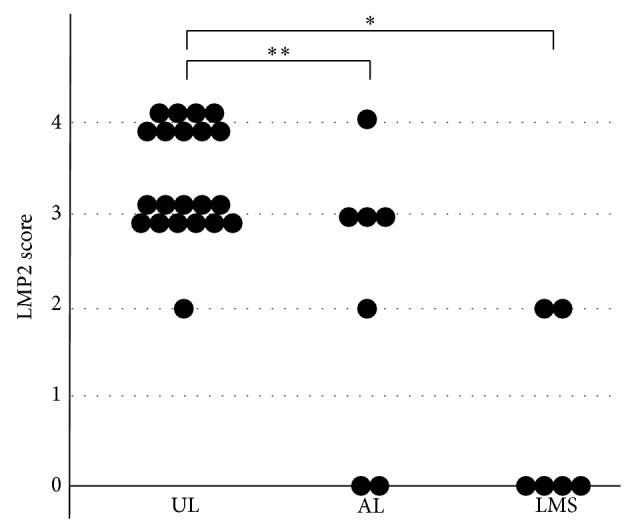
LMP score in SMTs. A significant difference was found in the LMP2 score for UL and AL and for UL and LMS (^*^ *P* < 0.01, ^**^ *P* < 0.05).

**Figure 3:**
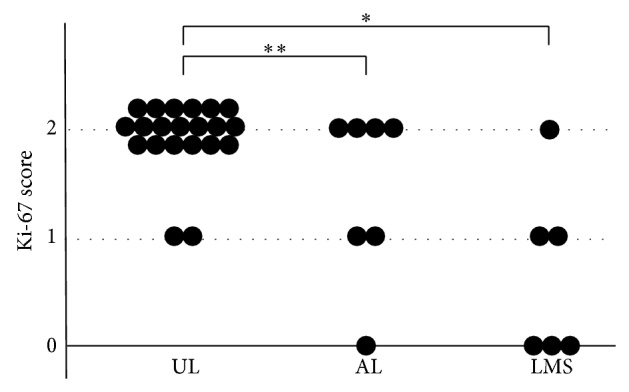
Ki-67 score in SMTs. A significant difference was found in the LMP2 score for UL and AL and for UL and LMS (^*^ *P* < 0.01, ^**^ *P* < 0.05).

**Figure 4:**
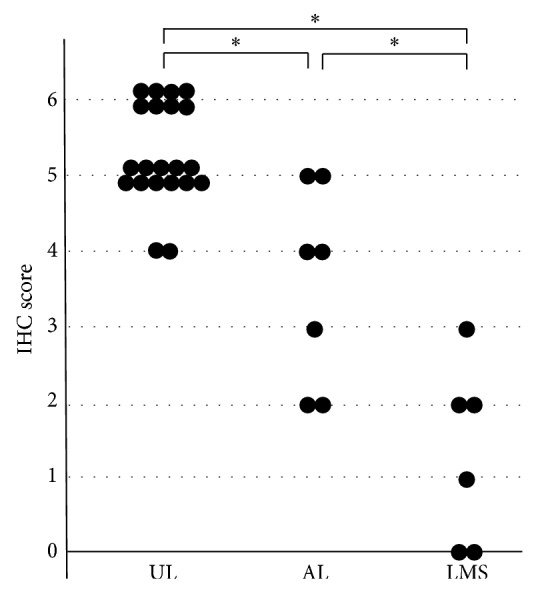
IHC score in SMTs. A significant difference was found in the LMP2 score for UL and AL, for UL and LMS, and for AL and LMS (^*^ *P* < 0.01).

Figure 1 shows the immunochemical staining results for LMP2 and Ki-67. The results of the IHC studies are shown in Figures 2–4.

The LMP2 score for UL was 2 to 4 points (median, 3 points), that for AL was 0 to 4 points (median, 3 points), and that for LMS was 0 to 2 points (median, 0 points). A significant difference was found in the product score of UL and LMS (*P* < 0.01) and of UL and AL (*P* < 0.05) (Figure 2).

The Ki-67 score for UL was 1 to 2 points (median, 2 points), that for AL was 0 to 2 points (median, 2 points), and that for LMS was 0 to 2 points (median, 0.5 points). A significant difference was found in the product score of UL and LMS (*P* < 0.01) and of UL and AL (*P* < 0.05) (Figure 3).

The IHC score for UL was 4 to 6 points (median, 5 points), that for AL was 2 to 5 points (median, 4 points), and that for LMS was 0 to 3 points (median, 1.5 points). A significant difference was found in the product score of UL and LMS, of UL and AL, and of AL and LMS (*P* < 0.01) (Figure 4). All cases of UL scored >4 points, and all cases of LMS scored <5 points. With respect to differentiation between AL and UL, the accuracy of inspection was the best when the cut-off score was set at 4 points: the sensitivity was 71.4% (95% CI, 41.8–88.5%) and the specificity was 90.5% (95% CI, 80.6–96.2%). With respect to differentiation between LMS and AL, the accuracy of inspection was the best when the cut-off score was set at 2 points: the sensitivity was 83.3% (95% CI, 54.4–95.3%) and the specificity was 71% (95% CI, 46.3–82.8%).

**Figure 5:**
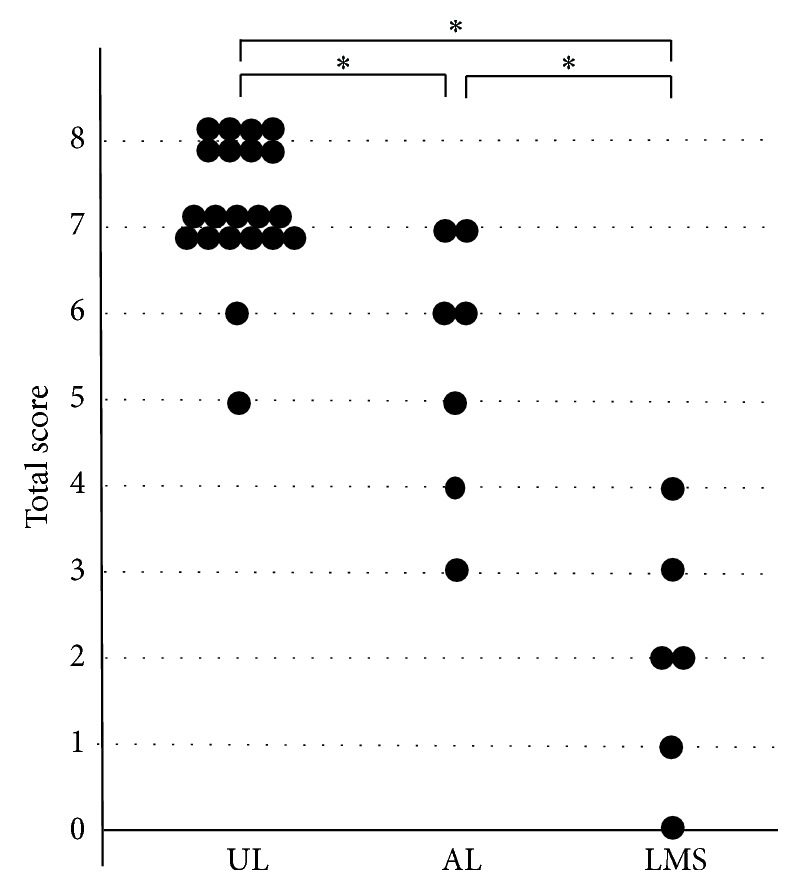
Total score in SMTs. A significant difference was found in the LMP2 score for UL and AL, for UL and LMS, and for AL and LMS (^*^ *P* < 0.01).

The total scores are shown in Figure 5. The total score for UL was 5 to 8 points (median, 7 points), that for AL was 3 to 7 points (median, 6 points), and that for LMS was 0 to 4 points (median, 2 points). A significant difference was found in the product score of UL and LMS, of UL and AL, and of AL and LMS (*P* < 0.01). With respect to differentiation between AL and UL, the accuracy of inspection was the best when the cut-off score was set at 6 points: the sensitivity was 71.4% (95% CI, 41.8–88.5%) and the specificity was 90.5% (95% CI, 80.6–96.2%). These results were the same as the IHC scores. With respect to differentiation between LMS and AL, the accuracy of inspection was the best when the cut-off score was set at 3 points: the sensitivity was 83.3% (95% CI, 54.4–95.3%) and the specificity was 85.7% (95% CI, 60.9–96.0%). The total score was more accurate than the IHC score.

## 4. Discussion

Statistically significant differences were found between UL and LMS and between UL and AL using one measure of IHC in the present study. However, there was no significant difference between AL and LMS. The combination of IHC findings and clinical features enabled detection of significant differences between AL and LMS.

The most important modality for preoperative diagnosis of SMTs is MRI. MRI findings suggestive of LMS include large heterogeneous masses and hemorrhage or cystic necrosis [11]. However, these findings are often present in cases of cellular leiomyoma, degenerated leiomyoma, and atypical leiomyoma. Recent reports have shown that the signal intensity on diffusion-weighted images may be useful for the differential diagnosis of SMTs [12]. Goto et al. reported that the combined use of dynamic MRI and serum LD measurement was useful for the diagnosis of LMS and degenerated leiomyoma [13]. FDG positron emission tomography findings of LMS feature moderate to intense FDG uptake. However, UL rarely shows high FDG uptake and LMS rarely shows mild FDG uptake [6], making it difficult to distinguish SMTs.

We aimed to improve the preoperative diagnosis of SMTs using transcervical uterine tumor biopsy [7]. We performed this technique, transcervical needle biopsy, on over 600 patients from 1994. No major complications, such as infection, intraperitoneal hemorrhage, or an injury of adjacent structures that required surgery, were reported to have occurred. For prevention of infection, we injected antibiotics during transcervical needle biopsy. To avoid injury of adjacent structures and major vessels, we perform transcervical biopsy carefully under transabdominal ultrasonography guidance [14]. Biopsy enables direct evaluation of tumor tissues. However, the diagnosis may be underestimated because the biopsy specimens are very small. Diagnosis of LMS requires two of the three following pathological features: cytologic atypia, a high MI, and CTCN. Small specimens sometimes cannot contain all features. CTCN is often confused with ischemic-type necrosis, and diagnosis of CTCN is difficult. Lim et al. reported that when six pathologists reviewed the occurrence of CTCN in 34 cases of LMS, full agreement regarding the presence or absence of CTCN was reached in only 12 cases (7 were thought to show CTCN) [15]. To prevent sampling error, three and over pieces of biopsy core were sampled. Considering the malignancy of LMS, we checked pathological findings of biopsy specimens in all the areas of all the cores strictly. If we suspected malignancy a little, we recommended the operation to the patients. In more detail, we think of the possibility of the malignancy, when there are some mild atypical nuclei (moderate to severe atypia which is one feature of LMS), less than 10 mitotic figures in all areas, or necrosis which cannot be clearly diagnosed as hyaline necrosis. On the other hand, because of that reason, we cannot exclude some patients of false positive.

Several studies have reported the IHC findings in SMTs. In LMS, the proliferative index of cells (Ki-67, PHH3) is often high and cell cycle regulatory proteins (p16, p53, p27, p21, and cyclins A and E) are often highly expressed. The expression of these markers is sometimes increased in cases of AL [16–19]. We herein reported the IHC staining results of Ki-67 in SMTs. For differentiation between LMS and UL, the best cut-off LI for Ki-67 with biopsy specimens was 5%. In the IHC evaluation of Ki-67 using surgical specimens, all LMS specimens showed an LI of ≥15%. Therefore, we classified the Ki-67 LI as 5% and 15% [8]. In the present study, the expression of Ki-67 was high in LMS, but there was no difference between AL and LMS.

Hayashi et al. reported the occurrence of LMP2 deficiency associated with the development of LMS. In IHC examination of LMP2 in resected specimens, 46 of 55 cases of LMS were negative, while all 48 cases of UL were positive [20]. The correlation between defective LMP2 function and uterine LMS tumorigenesis is not clearly understood. However, LMP2 has been suggested to play the role of a tumor suppressor by inducing reduced expression of interferon regulatory factor 1 and calponin 1 [21]. Likewise, the present study revealed differences in the staining pattern between UL and LMS in biopsy specimens (Figure 2). The combination of IHC analyses of Ki-67 and LMP2 was helpful for discrimination between LMS and AL.

SMTs have similar presenting symptoms including dysmenorrhea, menorrhagia, anemia, and pelvic masses. Serum levels of total LD in patients with LMS are often abnormally high, but those in patients with degenerated myoma and certain types of leiomyoma are also often elevated [13]. We examined which clinical indicators (chief complaint, MRI findings, serum LD levels, and menopause) are the most useful for the diagnosis of SMTs. Seven patients with AL and eight with LMS were analyzed, and significant differences in the serum LD level and menopause status were noted between patients with AL and those with LMS. The best cut-off LD ratio was 1.2; thus, we defined a high level of LD as >1.2 in this study. Most patients with LMS were postmenopausal; premenopausal patients were assigned 1 point.

The degree of malignancy associated with AL is unknown; most patients undergo hysterectomy to exclude LMS. Ly et al. found that the risk of recurrence in patients with AL is low and that treatment for atypical leiomyoma by myomectomy is therefore a viable option for women of childbearing age; confirmation of pregnancy is also possible after this procedure [4]. Thus, discrimination between AL and LMS is important.

In conclusion, this study suggests that the accuracy of a preoperative diagnosis of SMTs may be improved using a combination of IHC and clinical findings. However, the sample size of this study was small, and more cases need to be examined to reach a definitive conclusion.

## Figures and Tables

**(a):** 

Score	0	1	2

Intensity	Negative	Weakly positive	Positive

Range	Negative	Focal	Diffuse

**(b):** 

Score	0	1	2

LI	≥15	>5, <15	≤5

**(a):** 

Score	0	1

LD ratio	>1.2	≤1.2

**(b):** 

Score	0	1

Menopause	After	Before

**Table 3:** Clinical features in SMTs.

	UL (*n* = 21)	AL (*n* = 7)	LMS (*n* = 6)
Age (median)	43	46	55
Menopause	1 (5%)	1 (14%)	5 (83%)
High level LD	0	0	3 (50%)
